# Aesthetic Evaluation of Digitally Reproduced Art Images

**DOI:** 10.3389/fpsyg.2020.615575

**Published:** 2020-12-11

**Authors:** Claire Reymond, Matthew Pelowski, Klaus Opwis, Tapio Takala, Elisa D. Mekler

**Affiliations:** ^1^Department of Psychology, Center for General Psychology and Methodology, University of Basel, Basel, Switzerland; ^2^FHNW Academy of Art and Design, Institute of Visual Communication, University of Applied Sciences and Arts Northwestern Switzerland, Basel, Switzerland; ^3^Empirical Visual Aesthetics Lab, Department of Basic Psychological Research and Research Methods, Faculty of Psychology, University of Vienna, Vienna, Austria; ^4^Department of Computer Science, Aalto University, Espoo, Finland

**Keywords:** expertise, digitized artworks, subjective aesthetic evaluation, VIMAP, color saturation, evaluation time

## Abstract

Most people encounter art images as digital reproductions on a computer screen instead of as originals in a museum or gallery. With the development of digital technologies, high-resolution artworks can be accessed anywhere and anytime by a large number of viewers. Since these digital images depict the same content and are attributed to the same artist as the original, it is often implicitly assumed that their aesthetic evaluation will be similar. When it comes to the digital reproductions of art, however, it is also obvious that reproductions do differ from the originals in various aspects. Besides image quality, resolution, and format, the most obvious change is in the representation of color. The effects of subjectively varying surface-level image features on art evaluation have not been clearly assessed. To address this gap, we compare the evaluation of digital reproductions of 16 expressionist and impressionist paintings manipulated to have a high color saturation vs. a saturation similar to the original. We also investigate the impact of viewing time (100 ms vs. unrestricted viewing time) and expertise (art experts vs. laypersons), two other aspects that may impact the perception of art in online contexts. Moreover, we link these dimensions to a recent model of aesthetic experience [the Vienna Integrated Model of Top-Down and Bottom-Up Processes in Art Perception (VIMAP)]. Results suggest that color saturation does not exert a major influence on liking. Cognitive and emotional aspects (interest, confusion, surprise, and boredom), however, are affected – to different extents for experts and laypersons. For laypersons, the increase in color saturation led to more positive assessments of an artwork, whereas it resulted in increased confusion for art experts. This insight is particularly important when it comes to reproducing artworks digitally. Depending on the intended use, increasing or decreasing the color saturation of the digitally reproduced image might be most appropriate. We conclude with a discussion of these findings and address the question of why empirical aesthetics requires more precise dimensions to better understand the subtle processes that take place in the perception of today’s digitally reproduced art environment.

## Introduction

**Figure 1:**
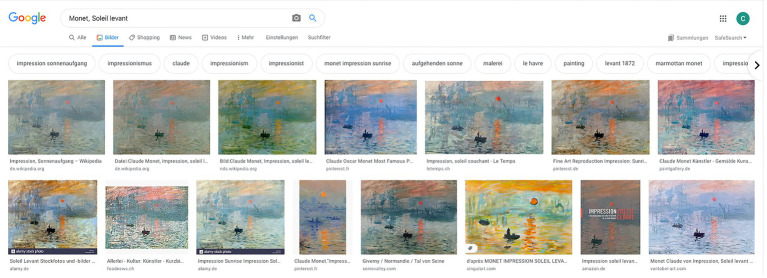
Google search “Monet, Soleil levant” (Accessed July 7, 2020).

Perceiving digitally reproduced images has become an indispensable part of our media-suffused everyday life. Digital reproductions specifically play a significant role in people’s encounter with works of art. For many viewers, their first encounter with an artwork does not take place in front of the physical artifact in a gallery or museum. Artworks are rather often first perceived as reproductions presented in a digitized form, selected, and compiled by a search engine on the Internet. Such reproductions resemble the original – more or less – and are often thought to be identical to the original artwork. But, digital reproductions of the same painting may differ in several ways, as shown in Figure 1: size, quality, resolution, and color can all vary considerably.

In digital reproductions, color is one of the image features expected to differ the most (Strickland et al., 1987; Eschbach and Kolpatzik, 1995; Yang and Rodriguez, 1995; Hu, 2007), especially in web-based presentations of art (see Figure 1). According to recent models of art processing (e.g., Leder et al., 2004; Graf and Landwehr, 2015; Pelowski et al., 2017; for an overview, see Pelowski et al., 2016), the color of an image may play a significant role in how art and its online reproductions are experienced. These surface-level features are among the first aspects viewers perceive upon encountering an artwork, are processed in a largely bottom-up fashion, and shape all subsequent stages of the aesthetic experience. Color saturation, in particular, stands out as one of the key components. As one of the three primary dimensions of the human experience of color, along with hue and brightness (Palmer and Schloss, 2015), saturation is processed during the first few milliseconds upon perceiving an image, and therefore likely impacts both our initial assessment – is it beautiful, do I like it? – and all following cognitive and affective processes. This raises the question of whether viewers of digitally reproduced images notice a subjective difference in color saturation, and to what extent this affects the aesthetic assessment of art images viewed online.

To date, only a few studies have investigated the influence of color saturation on the liking of digitized images of art. To address this gap, we provide empirical evidence of the relation between color saturation, expertise, and viewing time. The present study compares high-quality digital color reproductions of impressionist and expressionist works with matching versions of the same paintings with increased saturation. The effect of color saturation is assessed with regard to both general hedonic liking and various aesthetic outcomes tied to later processing stages (Pelowski et al., 2017). Based on the existing studies on the aesthetic evaluation of art (e.g., Leder et al., 2004; Pelowski et al., 2017) and, more specifically, on digitized reproductions of art (e.g., Locher and Dolese, 2004; Siri et al., 2018), we present the results of a quasi-experimental study of 75 art experts and 72 comparably inexperienced viewers of art.

In our results, we demonstrate that color saturation does not exert a major influence on liking. Cognitive and emotional aspects (interest, confusion, surprise, and boredom), however, are affected. As such, our findings suggest that the evaluation of more specific aesthetic reactions is needed to clearly depict the effect visual variables may have on the evaluation of digitized art images. Moreover, we show that color saturation affects viewers differently depending on their expertise in the field of art. While laypersons are positively influenced by increased color saturation, high saturation has a negative influence on experts’ evaluations. Also, and in contrast to previous studies (e.g., Kirk et al., 2011; Commare et al., 2018), we show that art experts are less consistent in their aesthetic judgments compared to laypersons. In particular, if they have time to do so, art experts tend to review their initial judgments given after viewing an artwork for a restricted period of time.

Investigating the effect of color saturation in digitized images on the evaluation of art may enable us to further highlight differences in the assessment processes of art experts and laypersons. Furthermore, our findings may encourage art museums and galleries to carefully adjust the color saturation of digitized art images they use on websites or as merchandising products.

## Related Work

In the following, we summarize existing research on the aesthetic processing of digitized art images. We also provide an overview of empirical research on color saturation, viewing time, and the relative expertise of the viewer, from which we derived the research questions for our study.

### Aesthetic Evaluation of Digitized Art

The differences between original artworks and different reproductions, including digitized art images, have been empirically studied (Berns, 2001; Locher et al., 2001; Locher and Dolese, 2004). Siri et al. (2018), for example, examined both the implicit sensorimotor and explicit cognitive responses of viewers when they observed artworks as originals in their physical form or as high-definition digital reproductions, both within a museum context and presented in the same size. Although there was no visible difference between originals and reproduced images in terms of physiological values, participants explicitly gave higher emotion scores to original artworks than to digital reproductions. In contrast, no significant differences were found with regard to participants’ judgment of color intensity and the aesthetic evaluation of digital and original works of art (Siri et al., 2018, p. 217). Locher et al. (1999) compared the perception of three different medial formats of the same artworks: originals, projected slides, and digital images viewed on a computer screen. They found that participants who viewed the reproduced images were aware that they were contemplating a reproduction and focused their attention on the performance and skills of the painter. Moreover, Locher et al. (1999, p. 128) reported that study participants directed their remarks almost exclusively to the art and not to the medium or to the interaction between art and medium. These results specifically show the ability of viewers to adapt to the medium an artwork is presented in Locher et al. (1999, p. 129), therefore, conclude that it is possible to designate “pictorial sameness” between originals and reproduced art images.

### Aesthetic Assessment and Color Saturation

When it comes to saturation preference in general, studies have shown that Western adults prefer more saturated colors over less saturated colors, provided the color is not “too vivid” (Granger, 1955, p. 15; see also Valdez and Mehrabian, 1994; Camgöz et al., 2002; Palmer and Schloss, 2015). These preferences lead to an increased attention to colored stimuli (Camgöz et al., 2004; Skulmowski et al., 2016). The perception of color can be described along three primary dimensions (Palmer et al., 2013): hue is characterized as the color’s tone, brightness as the lightness of the color, whereas color saturation refers to the relative purity or intensity of the color (Valdez and Mehrabian, 1994; APA Dictionary of Psychology, 2020).

#### Color Saturation in Digitized Images

Saturated colors are also preferred when it comes to the evaluation of digital images. Tinio and Leder (2009) investigated whether original or manipulated (including low sharpness, low saturation, and low contrast) digital photographs are preferred and showed that higher color saturation is perceived as a characteristic of higher image quality. Fedorovskaya et al. (1997) analyzed the colorfulness of digital images and demonstrated that slightly more colorful images were preferred compared to the original images (Fedorovskaya et al., 1997, p. 110).

The aesthetic quality of digital images affects the attractiveness of websites and subsequently influences viewer behavior (Hong et al., 2004; Li et al., 2014). Regarding the aesthetic evaluation of color on websites, Seckler et al. (2015) showed that blue hues and intermediately to highly saturated colors (together with low complexity and high symmetry) were most preferred. Contrary to this, Skulmowski et al. (2016) found that higher saturation did not lead to a greater preference in website evaluation. They claimed that, depending on the content of the website, color saturation had a negative effect. Skulmowski et al. (2016, p. 386) attribute this effect to the fact that saturated colors are characteristic of rather untrustworthy websites such as those of the yellow press, leading users to perceive very colorful websites as less credible. Reinecke et al. (2013, p. 7) offered a further differentiation by showing that education makes a difference in the assessment of color: “Participants with a doctorate were most negatively affected by high colorfulness, although participants with a higher education preferred websites with a similarly low colorfulness.”

#### Color Saturation and the Aesthetic Evaluation of Art

Models of aesthetic perception (e.g., Leder et al., 2004; Pelowski et al., 2017) describe a rapid initial perceptual analysis during which we perceive, process, and perhaps integrate the surface properties of an image such as color into our general reactions (Seckler et al., 2015; Skulmowski et al., 2016). The color saturation of an artwork can be understood as a primary influencer of the initial and subsequent “continuous affective evaluation” inherent to processing art (Leder et al., 2004, p. 492) and thus often directly influencing final evaluations (Arnheim, 2000; Schloss and Palmer, 2011; Nascimento et al., 2017). Pownall and Graddy (2016) found that the saturation of color significantly increases the price an artwork achieves at an auction. By contrast, studies that have manipulated the lighting of art images (Boust and Ezrati, 2006; Pelowski et al., 2019), which changes the perceived color temperature of an artwork, revealed no difference in aesthetic evaluation.

Due to the mixed evidence, it is unclear to what extent saturation – a feature that can be easily changed in digitally reproduced art images – affects aesthetic evaluation. It is, thus, not yet possible to sufficiently explain the influence of saturation on either the initial assessment or subsequent cognitive and emotional processing of digitized art images.

### Aesthetic Evaluation and Time

According to cognitive models of the perception of art (Leder et al., 2004; Pelowski et al., 2017), short-time elaborations take place within the first stage of aesthetic perception and consist of processing surface-level properties of the image as a purely bottom-up visual analysis. A deeper and top-down elaboration of the content and meaning of an image then arguably follows with a longer perception time.

#### Comparing Short vs. Long Viewing Times

Studies have shown that aesthetic judgments are made very quickly (Verhavert et al., 2018). Research on the aesthetics of websites has revealed that users evaluate the attractiveness of a website within the first 50 ms of encounter and that this rapid evaluation remains consistent over longer perception times (Lindgaard et al., 2006; Tuch et al., 2012; Skulmowski et al., 2016).

According to models of aesthetic perception (Leder et al., 2004; Pelowski et al., 2017), the time an art image is presented should play a role in evaluation, but the effect may only become apparent in later phases of the cognitive and top-down evaluation of the image and would not be discernible during the bottom-up stages of perception (Pelowski et al., 2017). In an examination designed by Smith et al. (2006), participants were presented a work of art for 1 s. Participants remarked afterward that they had barely noticed the image they had seen so briefly. But, bipolar scale-based ratings (e.g., pleasant/unpleasant or simple/complex using the semantic differential technique; Osgood, 1964) from this short viewing time did not significantly differ from ratings with a longer viewing time. Augustin et al. (2008) suspected that even a 50 ms viewing time would be sufficient to become aware of the content of an art image.

### Aesthetic Evaluation and Expertise

There is evidence that experts and laypersons differ in their aesthetic judgments (Eysenck, 1972; Winston and Cupchick, 1992; Reinecke et al., 2013; Weichselbaum et al., 2018). “Extensive training (or lack thereof)” (Leder et al., 2019, p. 111) in contemplating, questioning, and creating images exerts an influence on assessing artworks.

#### Consistency in Aesthetic Evaluation

Recent studies have investigated the consistency of expert judgments. Commare et al. (2018, p. 388) investigated the perceived complexity of artworks in laypersons and art experts. Their results showed that experts were far more consistent in assessing perceived complexity than laypersons when asked to evaluate the complexity of an artwork at two different times. Kirk et al. (2011) investigated the influence of sponsorship on subjective preferences for paintings. They showed that art expertise mitigated the influence of monetary favors in evaluating works of art. In comparison, judgments made by laypersons were favorably influenced by sponsoring, whereas experts’ judgments were more consistent with their personal judgments.

#### Expertise and Viewing Time

Expert and lay judgments are affected by viewing time. Höfel and Jacobsen (2003) showed that laypersons’ evaluation of beauty remained consistent over a few days, but this stability decreased with increasing time. According to models of aesthetic perception (Leder et al., 2004; Pelowski et al., 2017), viewers’ prior knowledge and expertise impact the cognitive evaluation of an image but hardly play a role in the early stages of perceiving it. By contrast, a recent study by Pelowski et al. (2020) suggests that differences between laypersons and experts already occur at the level of bottom-up processing. They found that persons with greater knowledge of art-like kitsch paintings (which were designed to have bright, highly saturated colors) less when they perceived them for 500 and 6,000 ms compared to when they initially saw them for 100 ms. Experts liked colorful pictures less over longer durations presumably because they switched from focusing on low-level color features to more historically or contextually based assessments. Interestingly, the differences in evaluation between laypersons and experts were already apparent in the 100 ms observation condition.

### Aesthetic Reactions Beyond Liking

The target of most studies in the field of empirical aesthetics is to focus on measuring hedonic responses. For example, the model outlined by Leder et al. (2004, p. 492) describes the outcomes of perceiving an artwork as an “aesthetic judgment,” which is typically manifested in questions concerning how much an artwork is liked or its relative beauty or quality, all of which often show a high correlation. But there are other possible targets beyond this. Leder also notes “aesthetic emotions,” which are often assessed *via* considerations based on basic circumplex models (Russell, 1980) of positive or negative valence or arousal. In assessing the impact that color saturation, viewing time, and expertise might have on judging digital art reproductions, there are also several specific aesthetic reactions that may be empirically observed.

#### The Vienna Integrated Model of Top-Down and Bottom-Up Processes in Art Perception

The Vienna Integrated Model of top-down and bottom-up processes in Art Perception (VIMAP) proposed by Pelowski et al. (2017) expands on the model of Leder et al. (2004) and further differentiates how the process of aesthetic perception results in distinct experiential outcomes. VIMAP offers a differentiated model of the behavioral, cognitive, and emotional reactions evoked by perceiving art. Five possible outcomes are proposed and can be circumscribed as follows: Outcome 1 is characterized as a low arousal state and evokes little emotion. Outcome 2 consists of an experience of novelty that can result in pleasure, confusion, or even feelings of sublimity and awe. Outcome 3 is when the viewer experiences a sense of flow, harmony, and emotional resonance, whereas in Outcome 4, negative feelings, including anger, shame, and sadness, are more salient. Outcome 5 denotes a transformative experience accompanied by feelings of epiphany, catharsis, and awe. Following these outcomes, it seems that questions such as “Is the image interesting for you?,” “Are you touched by the image?,” or “Does the image confuse you?” need to be asked in order to differentiate the complex reactions to an image. We, therefore, defined six items for our study that query one characteristic emotional or cognitive dimension of viewing art using the Aesthetic Emotions Scale (Aesthemos; Schindler et al., 2017). Aesthemos (Schindler et al., 2017) was developed in order to measure manifold reactions to art, film, literature, music, and other art forms with a focus on emotional reactions.

According to VIMAP, the emotional and cognitive elaboration of an artistic stimulus occurs at a later stage than the purely visual evaluation of the stimulus that happens within the first 100 ms of perception. The influence of color saturation on emotional and cognitive reactions to an image should thus be assessed only when the image is processed top-down and can be perceived for an unlimited time. With regard to the relative expertise of the viewer, models of aesthetic perception (Leder et al., 2004; Pelowski et al., 2017) offer little indication of whether expertise or a lack thereof may exert an influence on aesthetic evaluation at particular levels of art perception.

### Summary and Research Questions

Digitally reproduced art images are said to be similar to the originals, especially when it comes to specific aesthetic qualities of the image like color intensity, yet a difference is visible in terms of emotional reactions (Locher et al., 1999; Siri et al., 2018). Previous research has shown that higher saturated digitized images are preferred over less saturated ones (Tinio and Leder, 2009). Moreover, color – as a basic cue in visual perception (Palmer and Schloss, 2015) – has a strong impact on whether viewers like art stimuli (Pelowski et al., 2017). As a further factor, the time a visual artifact is perceived influences how it is evaluated (Pelowski et al., 2017). In addition, the level of expertise may modulate the assessment of an artwork (Commare et al., 2018), but this modulation may differ according to the level of processing (Pelowski et al., 2017). Based on previous research (Camgöz et al., 2004; Reinecke et al., 2013; van Dongen and Zijlmans, 2017), we argue that the augmented saturation of digitized reproductions of paintings will increase liking and show an influence on the general variety of outcomes as theorized by VIMAP. This effect should differ between laypersons and art experts. Our research questions can be condensed as follows:

Compared to laypersons, we expect art experts to be less influenced by the manipulation of the image surface (i.e., color saturation), whereas laypersons’ liking of digitized images will be influenced by color saturation.

Moreover, the effect of color saturation on liking will be more strongly influenced by viewing time in the case of experts than in the case of laypersons, as predicted by models of the cognitive processing of aesthetic stimuli (Leder et al., 2004; Pelowski et al., 2017).

We further expect that the manipulation on color saturation will be more visible with regard to specific emotional reactions (“boredom,” “interest,” “insight,” “confusion,” “being moved,” and “surprise”) than with regard to liking.

## Materials and Methods

The study followed a quasi-experimental mixed-subject design. The independent variables were saturation (original vs. manipulated saturation), expertise (art experts vs. laypersons), and viewing time (100 ms vs. unlimited).

### Participants

Seventy-two psychology students from the University of Basel [56 female, 15 male, and 1 preferred not to answer; *M_age_* = 23.24, *SD* = 5.27, and range = 19–50; all lay viewers of art as assessed *via* post study interviews the Vienna art interest and art knowledge questionnaire (VAIAK); Specker et al., 2020] and 75 art-history students from the University of Basel and the Academy of Art and Design at the University of Applied Sciences and Arts Northwestern Switzerland (47 women, 25 men, and 3 preferred not to answer; *M_age_* = 27.25, *SD* = 7.42, and range = 18–62; considered to be relatively expert viewers of art) participated in the experiment. Participants were asked to inform the experimenter about any vision impairments that were not corrigible to normal with glasses or contact lenses. Participants were also asked to inform the experimenter if they had an abnormal color vision. One participant stated that he could only see with one eye and was therefore excluded from the analysis.

Participants were compensated with course credit or monetary compensation (about USD 15). All participants were asked to provide signed informed consent and were informed that they could quit the study at any time and that all data collected in the study would be evaluated anonymously. The study was conducted in accordance with the ethical guidelines of the University of Basel.

### Stimuli

The stimuli consisted of 16 high-quality digital color photographs (100 dpi) of paintings from the impressionistic and expressionistic periods of the beginning of the twentieth century, including landscape pictures, portraits, still lives, and groups of figures (see Supplementary Table A1 for a full list of the artworks, artists, and links to the retrieved paintings). Nine of the original paintings were in the possession of the Kunstmuseum Basel and were downloaded from the online collection of the museum’s website.^1^ The remaining seven paintings were from other art museums or private collections and were downloaded from the website of the respective museum or auction house. As such, these images were expected to represent the authoritatively most faithful reproductions of the original paintings’ contrast and color saturation.^2^

**Figure 2:**
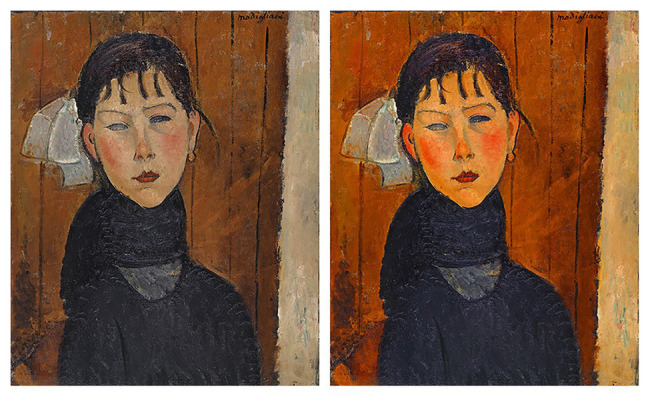
Example of an art image with saturation matching the original and with increased saturation. On the left, the photograph of the painting *Marie* by Modigliani (1918), as it was presented in the online collection of the Kunstmuseum Basel. On the right, the same image with a 60% increase in saturation.

Expressionistic and impressionistic paintings were selected because these styles are known for using color as a formative pictorial element (Alscher, 1968). We deliberately chose original artworks that feature a muted color palette and low color saturation (see Figure 2 for an example). The fact that high saturation is usually recognized as a distinguishing feature of impressionistic and expressionistic style (Alscher, 1968) allowed us to increase color saturation in a way that would not appear overly artificial or incongruous to art experts (Boust et al., 2006).

In addition to the original versions of the 16 images, we also created a matched set of the same 16 paintings but with increased color saturation (leading to a final stimulus set of 32 images). Both the original and manipulated images were scaled to have an identical height of 800 px. Figure 2 shows a digital photograph of an original artwork and an example of the results of the image manipulation procedure.

All of the manipulations were performed by the first author using Adobe Photoshop CC (version 19.1.4, www.adobe.com) and conducted individually for each image. In consultation with the research team, saturation values were increased between 50 and 80% linearly on the whole image (see Supplementary Table A2 for more detailed information on the manipulations). This range was chosen to ensure that the changes to color saturation were *clearly visible* at first glance but were also moderate enough to avoid overly colorful-looking images, which risk being perceived as garish (Fedorovskaya et al., 1997). Note that we were specifically interested in viewers’ *subjective* responses rather than assessing objective colorimetric thresholds of the saturation level.

### Procedure

Participants (psychology students, hereafter “laypersons,” and art-history or design students, hereafter “experts”) viewed the images in a lab setting, ensuring that they saw the images under the same monitor settings for color and brightness and the same ambient lighting. Five computers were used for the study, arranged in such a way that participants could not see what was on the other screens. The screens of all five computers were uniformly calibrated (using the Apple Display Calibrator Assistant). The brightness of the screens was set to maximum and the automatic adjustment to brightness was switched off. The ceiling lighting always switched on during the experiment. No other light sources were present, except two ceiling windows, which prevented direct light from entering the room. The experiment was programmed in Unipark (2017) and presented on 21.5 in iMac monitors (resolution: 1,920 × 1,080 px, 19.541 × 18.730 in). Viewing distance was about 23.7 in, resulting in a visual angle of ~48°.

Before starting the experiment, participants were requested to give informed consent, to provide demographic information, and to confirm that they had understood the instructions. Moreover, participants were instructed to indicate after completion of the study whether they had recognized any of the images and if so, which ones. However, less than five participants noted that at least one of the images appeared familiar to them. Hence, we did not pursue this further. The study duration was about 30 min on average.

The experiment consisted of two blocks (blocks 1 and 2) presented in the same order for all participants and followed by an art questionnaire. Pilot tests indicated that evaluating the complete set of 2 × 16 images was too taxing, so participants were randomly assigned to one of the two different sets of 16 total images (containing eight originals and manipulated versions of the same paintings). The presentation order of the stimuli was fully randomized. Participants were not informed that image saturation had been manipulated.

#### Block 1: 100 ms Viewing Time and Liking

**Figure 3:**
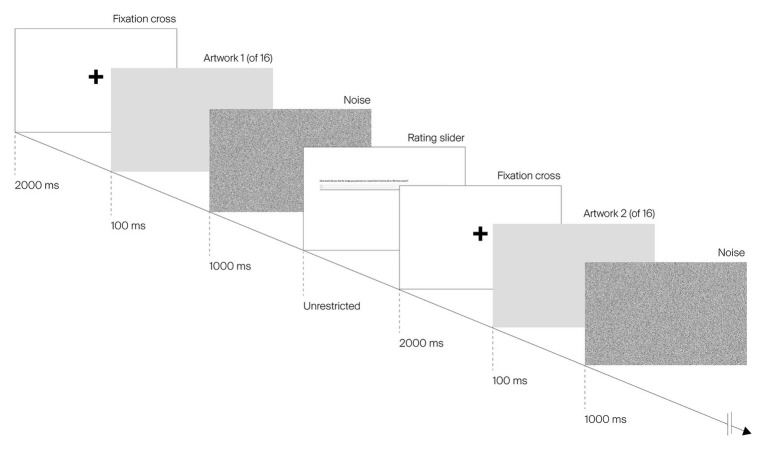
Example sequence of image presentation for block 1 (100 ms viewing time) of the study. Images are cropped for legibility purposes.

Participants were presented with each of the 16 images (in addition to one repeated image shown twice to measure the test-retest reliability; see results). Each image was shown on its own for 100 ms (Pelowski et al., 2017, 2020), centered on a white background. As shown in Figure 3, each image was preceded by a fixation cross on a white background for 2,000 ms and followed by a black-and-white noise mask for 1,000 ms. After the noise mask, a slider was displayed on the screen, asking participants to indicate how much they liked the image. Participants were instructed to note their first impression of the image and to indicate their liking as quickly as possible so as to assess their initial response to the image’s low-level visual features (i.e., its saturation). This procedure was repeated for all images in the set.

#### Block 2: Unlimited Viewing Time, Liking, and Specific Aesthetic Reactions

The same 16 images were shown for an unlimited period of time. Again, each image was shown on its own, centered on the top of the screen. Participants were instructed to take their time and to view each painting for as long as they wished. Upon scrolling down, participants had access to the questionnaire. Participants could thus continue viewing the image while answering the questions. Participants again indicated their liking on a rating slider and clicked a “Next” button to access the next image.

### Measures

We collected measurements on three different scales during the three phases of the experiment. Blocks 1 and 2 contained a liking slider to rate the stimuli presented. In addition to the liking slider, 12 items to measure six dimensions of the aesthetic evaluation were presented in block 2. During the third phase and before the study was completed, art expertise was assessed.

#### Liking

To measure participants’ liking of an artwork on blocks 1 and 2, a rating slider was displayed below the image offering the possibility to rate the digitized paintings from 0 to 100 (0 = “not at all,” 100 = “very much”).

#### Specific Aesthetic Reactions

In block 2, in addition to the liking slider, 12 items [5-point Likert-type scale ranging from “not at all” (1) to “very” (5)] from the Aesthemos (Schindler et al., 2017) were displayed in randomized order under the image. Two items for each of the following six dimensions were defined: “being moved,” “boredom,” “confusion,” “insight,” “interest,” and “surprise.” These dimensions were chosen as they reflect the five outcomes described in the VIMAP (Pelowski et al., 2017). “Boredom” resembles outcome 1 (“facile, default”), “surprise” corresponds to outcome 2 (“novelty, insight”), “being moved” is considered a characteristic of outcome 3 (“harmony, flow, emotional resonance”), “confusion” reflects outcome 4 (“negative, abort”), and “insight” corresponds to outcome 5 (“transformation”). The dimension of “interest” was not attributed to a specific outcome posited by VIMAP, as it is argued to occur on different outcome levels of the model. Yet, it was included to measure a basic reaction to art (Silvia, 2013). Items were reworded into present tense (from past tense in the original Aesthemos questionnaire), as participants were asked to rate their feelings upon viewing the image.

#### Art-Expertise Questionnaire

Finally, after completing the study, participants were asked to list as many painters as they could name within 60 s, a technique which has been previously employed (e.g., Krauss et al., 2019) to provide a quick estimation of relative art expertise or knowledge. Additionally, participants rated their interest in art *via* the 11-item “interest” battery from the VAIAK (Specker et al., 2020).

## Results

All participants completed all portions of the study. The test-retest reliability, as measured by the repeated images in block 1, showed that all image ratings had good reliability (total *r* = 0.884, *p* < 0.001, *n* = 147). Thus, all data were retained for analysis. For all statistical tests throughout the paper, we used an alpha level of 0.05. Due to the exploratory nature of the study, no adjustments for multiple comparisons were performed.

As expected, VAIAK (Specker et al., 2020) scores indicated that experts (art-history and design students) scored significantly higher on art interest (*M* = 5.345, *SD* = 0.963, range = 2.18–6.82) than laypersons [*M* = 3.107, *SD* = 0.962, range = 1–5.36; independent sample *t*-test *t*(145) = 14.094, *p* < 0.001]. Experts were also able to list significantly more painters within 60 s (*M* = 6.45, *SD* = 3.189, range = 1–14) than laypersons [*M* = 4.54, *SD* = 2.222, range = 1–12; *t*(140) = 4.089, *p* < 0.001].

### Impact of Saturation and Expertise on Image Liking for 100 ms and Unlimited Viewing Times

To evaluate the effects of saturation, expertise, and viewing time on image liking, a repeated-measures ANOVA was calculated. Saturation (original vs. manipulated) × viewing time (100 ms vs. unrestricted) were defined as within-participant factors and expertise (experts vs. laypersons) as a between-participant factor. Supplementary Table A3 lists descriptive statistics for each image per viewing time and color saturation conditions.

**Figure 4:**
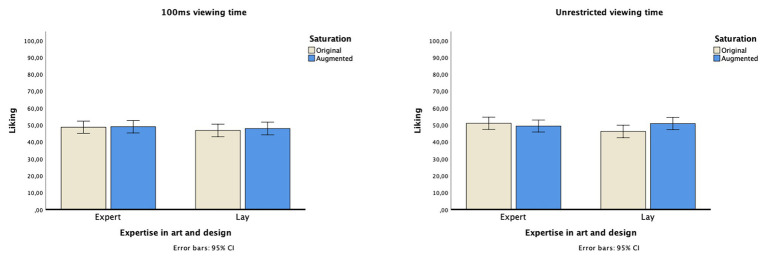
Mean liking for the eight conditions (saturation: original vs. manipulated, viewing time: 100 ms vs. unrestricted, and expertise: experts in art or design vs. laypersons).

A significant main effect was detected for saturation [*F*(1,145) = 3.995, *p* = 0.047, *η*^2^*_p_* = 0.027], with more highly saturated images liked more than the original ones across all participants. No significant main effects were found for either expertise [*F*(1,145) = 0.436, *p* = 0.510, *η*^2^*_p_* = 0.003] or viewing time [*F*(1,145) = 2.860, *p* = 0.093, *η*^2^*_p_* = 0.019]. The results did reveal, however, a significant interaction between expertise × saturation [*F*(1,145) = 10.214, *p* = 0.002, *η*^2^*_p_* = 0.066]. Compared to experts (original images: *M* = 49.835, *SD* = 14.463; manipulated images: *M* = 49.162, *SD* = 15.034), laypersons liked more saturated images (*M* = 49.382, *SD* = 14.856) over less saturated images (*M* = 46.460, *SD* = 15.172). Figure 4 displays mean liking across the eight conditions.

Moreover, we found a significant three-way interaction between saturation × viewing time × expertise [*F*(1,145) = 7.636, *p* = 0.006, *η*^2^*_p_* = 0.050]. *Post hoc* comparisons within the expertise groups showed that the effect on liking was driven by a significant saturation effect in the laypersons group [*F*(1,71) = 14.309, *p* < 0.001, *η*^2^*_p_* = 0.168] but not in the experts group [*F*(1,74) = 0.682, *p* = 0.412, *η*^2^*_p_* = 0.009]. Viewing time had no significant impact on laypersons’ liking [*F*(1,71) = 1.662, *p* = 0.202, *η*^2^*_p_* = 0.023] or on art experts’ liking [*F*(1,74) = 1.334, *p* = 0.252, *η*^2^*_p_* = 0.018].

#### Consistency in Aesthetic Judgments for Experts and Laypersons Over Different Viewing Times

To analyze the consistency of liking ratings between the very short (100 ms) and the unrestricted viewing time for original and manipulated saturation, we report correlations (Pearson product-moment) between the viewing times for each expert or layperson. Pearson correlation was chosen because image liking was rated on a continuous scale from 0 to 100 (Bortz, 2013). Liking correlations between 100 ms vs. unrestricted viewing times were high overall, indicating that liking ratings remained relatively stable. However, experts’ liking of original and manipulated images correlated less strongly between viewing times (*r*_original_ = 0.694, *p* < 0.001, *n* = 75; *r*_manipulated_ = 0.730, *p* < 0.001, *n* = 75) than laypersons’ liking (*r*_original_ = 0.831, *p* < 0.001, *n* = 72; *r*_manipulated_ = 0.835, *p* < 0.001, *n* = 72). Overall, both art experts’ and laypersons’ liking ratings were more consistent for the manipulated images.

**Figure 5:**
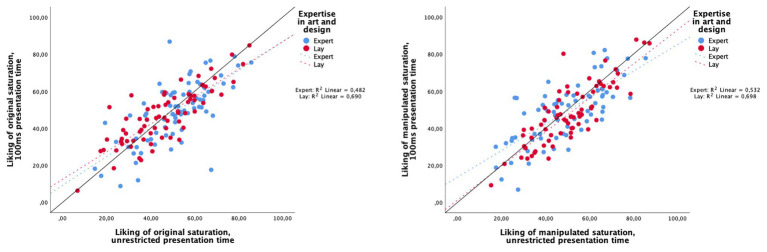
Correlation between image liking and viewing time – 100 ms (*y*-axis) and unrestricted (*x*-axis) – for experts and laypersons. Liking scores for original saturation are on the left and for manipulated saturation on the right.

To further visualize the relationship between liking ratings, Figure 5 displays the mean ratings for each participant (individual dots) between the 100 ms and the unrestricted viewing times (*y*- and *x*-axis, respectively) for both laypersons and experts (red and blue dots, respectively) and between the original saturation (Figure 5, left side) and the manipulated saturation (right side) conditions. Dots above the 45° line indicate that a participant reported higher image liking in the 100 ms condition; dots below the line indicate that participants reported higher liking in the unrestricted condition; those appearing exactly on the line were liked equally across conditions.

Mean ratings over all images show that laypersons were more consistent in their judgments across both viewing times (100 ms: *M* = 47.338, *SD* = 15.207; unrestricted: *M* = 48.504, *SD* = 15.089) than experts, who appeared to increase their liking rating, when there was more time to process the image (100 ms: *M* = 48.817, *SD* = 15.863; unrestricted: *M* = 50.180, *SD* = 14.522). An independent sample *t*-test revealed a significant difference between the two groups [*t*(145) = 0.1331, *p* = 0.042, *η*^2^*_p_* = 0.011]. Furthermore, experts took longer to view and evaluate the images in the unrestricted time condition (block 2). Experts spent around 62.44 s (median) per image, whereas laypersons only spent around 44.72 s (median). As requirements for a *t*-test were not met (variance homogeneity and normal distribution were violated), a nonparametric test was calculated: a Wilcoxon-Mann-Whitney test revealed a significant difference between the groups (*z* = −5.33, *p* < 0.001, *n* = 147), with an effect size of *r* = 0.44, which according to Cohen (1992) corresponds to a large effect.

### Saturation, Expertise, and Specific Aesthetic Reactions

**Figure 6:**
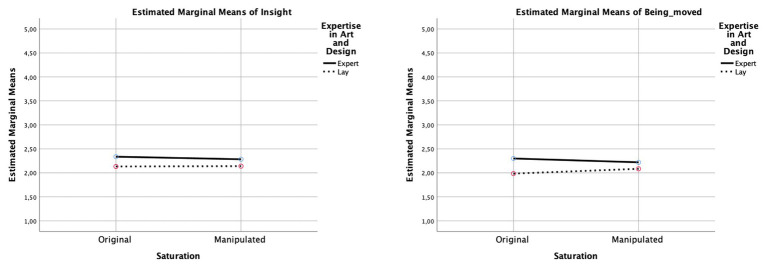
Interaction diagrams for “insight” and “being moved.” The diagrams show the direction of the effects for each dimension between the original color saturation and the manipulated color saturation, comparing experts and laypersons.

**Figure 7:**
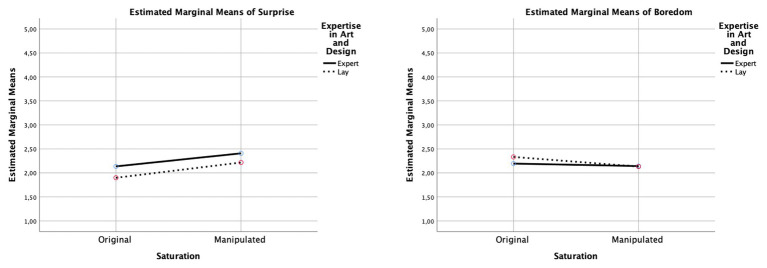
Interaction diagrams for “surprise” and “boredom.” The diagrams show the direction of the effects for each dimension between the original color saturation and the manipulated color saturation, comparing experts and laypersons.

**Figure 8:**
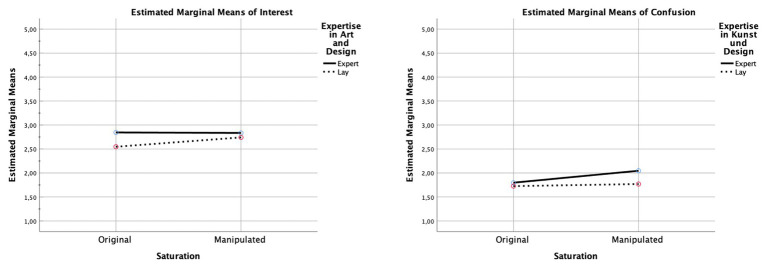
Interaction diagrams for “interest” and “confusion.” The diagrams show the direction of the effects for each dimension between the original color saturation and the manipulated color saturation, comparing experts and laypersons.

To study the effect of saturation in more detail, we investigated six dimensions of aesthetic reactions (block 2) that cover aesthetic assessments beyond basic liking. Table 1 shows the mean ratings for the six specific aesthetic reactions across all expertise and saturation conditions (see also Figures 6–8). To analyze the effects of saturation and expertise, repeated-measures ANOVAs (saturation, within-participant; expertise, between-participant) were calculated for each scale individually.

**Table 1:** Descriptive statistics for specific aesthetic reactions of art experts and laypersons, for original and manipulated saturation.

		Experts		Laypersons		All	
		(*n* = 75)		(*n* = 72)		(*n* = 147)	
Item	Saturation	*M*	(*SD*)	*M*	(*SD*)	*M*	(*SD*)
Insight	Original	2.34	(0.59)	2.13	(0.63)	2.24	(0.61)
	Manipulated	2.28	(0.66)	2.14	(0.62)	2.21	(0.64)
Being moved	Original	2.30	(0.66)	1.98	(0.58)	2.15	(0.64)
	Manipulated	2.22	(0.68)	2.08	(0.57)	2.15	(0.63)
Interest	Original	2.85	(0.66)	2.55	(0.61)	2.70	(0.65)
	Manipulated	2.84	(0.67)	2.74	(0.58)	2.79	(0.63)
Confusion	Original	1.80	(0.52)	1.73	(0.54)	1.76	(0.53)
	Manipulated	2.05	(0.68)	1.77	(0.55)	1.91	(0.64)
Surprise	Original	2.14	(0.57)	1.90	(0.56)	2.02	(0.57)
	Manipulated	2.41	(0.60)	2.22	(0.63)	2.31	(0.62)
Boredom	Original	2.19	(0.53)	2.33	(0.69)	2.26	(0.62)
	Manipulated	2.14	(0.55)	2.13	(0.62)	2.14	(0.58)

Item scores range from 1 to 5.

As listed in Table 2, significant main effects of saturation were observed for “surprise” and for “interest,” indicating that participants deemed more saturated images more surprising and interesting (see Figures 6–8). Similarly, saturation significantly affected “boredom” and “confusion,” suggesting that participants found increased-saturation images less boring and confusing. Significant main effects were also found for expertise on “surprise,” “interest,” “being moved,” and “confusion.” Experts not only reported more surprise and interest, and felt more moved, but also more confused.

**Table 2:** Results of the repeated measures ANOVA on saturation, expertise, and specific aesthetic reactions.

Saturation				Expertise			
Item	*F*	*η*^2^*_p_*	*p*	Item	*F*	*η*^2^*_p_*	*p*
Insight	0.507	0.003	0.478	Insight	3.177	0.021	0.077
Being moved	0.099	0.001	0.754	Being moved	5.327	0.035	0.022^*^
Interest	5.918	0.039	0.016^*^	Interest	4.037	0.027	0.046^*^
Confusion	13.745	0.087	0.000^**^	Confusion	4.007	0.027	0.047^*^
Surprise	69.582	0.324	0.000^**^	Surprise	5.575	0.037	0.020^*^
Boredom	11.934	0.076	0.001^*^	Boredom	0.493	0.003	0.484
Saturation × Expertise							
Item	*F*	*η*^2^*_p_*	*p*				
Insight	0.856	0.006	0.357				
Being moved	7.405	0.049	0.007^*^				
Interest	7.265	0.048	0.008^*^				
Confusion	6.782	0.045	0.010^*^				
Surprise	0.440	0.003	0.508				
Boredom	4.177	0.028	0.043^*^				

* Significant at *p* < 0.05;

** Significant at *p* < 0.001.

Moreover, significant saturation × expertise interactions were found for all dimensions, except “surprise” and “insight.” With regards to “interest,” laypersons found images with increased saturation more interesting, whereas experts found them less interesting. A similar pattern was observed for “being moved”: laypersons found images with increased saturation more moving, and experts felt less moved. In contrast, with regard to “boredom,” experts rated original and manipulated images to a similar degree, whereas laypersons found the original images more boring. Finally, concerning “confusion,” experts rated images with increased saturation as more confusing, perhaps because the colors did not match their expectations of the artistic style or of similar art, whereas, for laypersons, increased saturation did not have an effect.

## Discussion

An innumerable variety of digitized art images can be found online that may differ substantially from the original in terms of several features, even though they show the same image content and are attributed to the same artist. We conducted a study that reflects a similar encounter with digitally reproduced art. Our focus was to manipulate color saturation (using a matched condition of both high-fidelity versions and increased-saturation versions of the same paintings) – an image component that substantially varies in digital reproductions of art images on the Internet (Eschbach and Kolpatzik, 1995; Yang and Rodriguez, 1995) – and to examine the effect of this surface-level image feature on liking and more specific aesthetic reactions in lay and expert viewers.

### The Influence of Saturation on Aesthetic Judgments

We found a main effect for color saturation – more saturated images were liked relatively more when compared within-participants – that extended across both expertise levels and both during short and unrestricted viewing times. The results reflect earlier findings that saturated colors are preferred in general (e.g., Palmer and Schloss, 2015 on saturated colors in general; Seckler et al., 2015 on colors on websites) and underline the argument that the manipulation of saturation exerts an effect on the evaluation of an image. This finding is particularly important when it comes to reproducing art images digitally – which are then to be used, for example, in a virtual gallery or museum, in an online art catalog or as a merchandising material and souvenirs. As our study has shown, an increase in color saturation affects viewers differently depending on their expertise. Since laypersons seem to judge images primarily by their surface texture, increasing the color saturation has a positive effect on their assessment of an artwork. For art experts, who are used to working with images and who focus mainly on the content and meaning of a work, increasing the saturation has the opposite effect and can lead to confusion.

Our results may also further refine the difference between originals and digital reproductions. Although we used and investigated the aesthetic evaluation of two versions of digitized art images, our results provide more information on the aesthetic evaluation of digital art images. If “faithful high-quality digital reproduction of works of art could be as arousing as the original works of art” (Siri et al., 2018, p. 201), the color saturation of the digitally reproduced artwork must correspond exactly to the original in order not to influence the image’s appearance.

At the same time, and against our expectations, our results show that increased saturation had a quite small effect on liking. These results are in contrast to earlier investigations (van Dongen and Zijlmans, 2017) that demonstrated the effect of contrast as a surface-level manipulation on the evaluation of artworks. Perhaps the manipulation of contrast addresses a different level of processing than the manipulation of saturation. While image saturation and contrast are typically subsumed under the same processing level (perceptual analysis; Leder et al., 2004; Pelowski et al., 2017), they are distinct image properties that may have different effects on the liking of an artwork. It should also be noted that participants in our study only liked the images to a moderate extent. In other words, they did not have strong feelings about the images, and saturation manipulation only subtly affected their aesthetic judgments. It remains to be seen to what extent saturation manipulation would impact aesthetic processing for images that viewers strongly like or dislike.

Our findings also differ from studies on the effects of color saturation on the evaluation of websites, which have shown the manipulation of saturation to possess a strong effect (Seckler et al., 2015). It may be argued that art images require a more nuanced and elaborate evaluation than webpages, which might explain the differences in results. Nevertheless, our findings are more in line with the results of Boust and Ezrati (2006), who found that although different lighting conditions alter the color appearance of artworks, viewers’ assessment of artworks remained consistent across different color conditions. Boust and Ezrati (2006, p. 6) argue that this may be due to “relational color constancy,” suggesting that the relation of colors within the painting is more influential than the absolute value of color. This lack of a substantial effect was also noted by Pelowski et al. (2019), who found very small effects from different color temperatures of lighting on the assessment of artworks.

### Effects of Expertise and Time

Differences between our results and previous studies of color saturation in digital images could be related to expertise. For example, Seckler et al. (2015) did not distinguish in their study between web-design experts and laypersons. In our study, although all participants tended to prefer saturated images in general, art experts were comparatively less influenced by manipulations of the image surface, whereas laypersons seemed more susceptible to the colors of an image when indicating how much they liked it. This result itself is in keeping with past studies on the influence of context (e.g., Kirk et al., 2011), which have shown that expertise might tend to insulate against large impacts on appraisals of art from alterations to the image surface. This might also be explained by the relative attention to both low-level surface features of art – including saturation – and more top-down, art-historical aspects, since experts potentially give more emphasis to the latter features when evaluating art (e.g., see Pelowski et al., 2020). Such a result was suggested by the three-way interaction between saturation, expertise, and time in the present study.

Our study also produced interesting findings with regard to viewing time. The lack of difference in liking ratings following both the 100 ms and open-ended viewing duration conditions (and in fact a high correlation between ratings at the level of individual viewers and individual artworks) supports the argument that saturation is one of the features of images that may be processed first, almost immediately following viewing an image. This is in line with the findings of Lindgaard et al. (2006) and suggests that a rapid assessment of visual artifacts not only applies to websites but also to digitized images of paintings when expertise in this field is low. In accordance with cognitive models of aesthetic perception (Leder et al., 2004; Pelowski et al., 2017), a very short viewing time affords only bottom-up perceptual analysis, in which surface-level properties of the image such as saturation are processed and assessments of the image content are not yet included.

Interestingly, one could argue based on our results that, despite its small effect size, saturation may have an impact when considered at the level of basic hedonic (i.e., liking) responses, which may not themselves change or may even inform subsequent analyses. Laypersons’ first impression of an image is in strong accordance with their liking of the artwork, even when there is enough time to contemplate and evaluate it. At the same time, as suggested above, our results also show that art experts are not quite as consistent in their liking of judgments in very short vs. unrestricted viewing times. This is in contrast to Commare et al. (2018), who found that experts exhibit more consistency in complexity judgments than laypersons. Our findings support the claim, predicted by the models of Leder et al. (2004) and Pelowski et al. (2017), that expertise affects the evaluation of an image, but only at a later (top-down) stage of processing. Our results suggest that when expertise is low, the assessment of an image at a later stage of processing is consistent with the first impression and the evaluation of the image’s surface-level characteristics. But if expertise and background knowledge in this area is more pronounced, it is activated at a later stage and revises the initial, bottom-up visual impression of the perceived image. This is further supported by the fact that experts took significantly more time than laypersons to view and evaluate the images in block 2. This suggests that experts revise their judgments when there is enough time to process the artwork and that they take their time to do so, potentially indicating that experts ground their evaluation more on the content of the image than on surface features, such as saturation. These results are in line with the study conducted by Pelowski et al. (2020), which found that experts might engage in more top-down processes to evaluate an image when there is more time available and thus might reassess low-level features.

Additionally, investigating the perceived visual complexity of the artworks may serve to further differentiate image processing between different levels of expertise and viewing times: for instance, visual complexity and colorfulness have been found to shape viewers’ first impressions differently, depending on their age and education level, respectively (Reinecke et al., 2013). Moreover, experts and laypersons perceive the complexity of images differently, with the former tending to appreciate higher perceived complexity more (Reber et al., 2004).

### Liking vs. Aesthetic Reactions

The present study supports previous theoretical arguments about aesthetic reactions or features of aesthetic experience beyond basic hedonic liking. As our results show, the effects of saturation manipulation only became apparent when participants were asked to rate their aesthetic reactions to the artworks. Alternatively, our findings reveal that aesthetic reactions indicating an outcome paraphrased by easy-to-achieve positive or also negative sensations on VIMAP showed significant effects. Interestingly, the specific dimensions we looked for in our study showed significant differences according to the manipulation of saturation, and they also revealed differences between experts’ and laypersons’ evaluations of the images. As shown in Figures 6–8, color saturation influenced various aesthetic reactions in laypersons, and they exhibited more differences in their ratings than experts. The dimension “confusion” revealed a crucial aspect: color saturation hardly influenced experts’ judgments regarding their interest or boredom while viewing the image and instead led to an irritation when the color intensity was augmented. Experts were more moved by the image, had more interest in it, and experienced a more pronounced sense of insight compared to laypersons. This indicates that, with regard to these aesthetic reactions, art experts cannot be swayed by surface manipulation of an image. Experts did, however, react to a change in color intensity by showing more confusion and surprise.

It should also be noted that the observed effect sizes were rather small. In line with the VIMAP stages of higher-order cognitive processing (Pelowski et al., 2017), we did not anticipate particularly pronounced differences for these dimensions, especially because we expected it to be unlikely that people experience such strong emotional reactions to digital reproductions of art images they see on a computer screen – particularly in a laboratory setting with a sequence of viewed artworks (Pelowski et al., 2017). This explains why both “being moved” and “insight” – which are attributed to outcomes 3 and 5 on the VIMAP, respectively, and are characterized by strong emotional responses and described with feelings of flow or transformation – were not affected by color saturation.

More generally, we argue that in studies of empirical aesthetics, the dimension of liking may not be precise and differentiated enough to adequately reflect and evaluate the perception of art images. In our study, the evaluation of liking could not reflect the diverse dimensions of perceiving an image that were affected by manipulating color saturation, neither in the perception of experts nor in that of laypersons. Including more differentiated and specific factors than liking gave a more detailed impression of the effect of color saturation on the perception of an artwork. We conclude that asking only about the liking of an artwork is not specific enough to evaluate responses to an art image. More research is needed to carefully examine the processes occurring in interaction with art and to analyze specific aesthetic reactions in detail.

### Limitations and Future Work

In the following, we address the main limitations of our work and discuss avenues for future work. First, our study employed only a small range of artistic styles (impressionism and expressionism) and a limited pool of viewers. Findings might differ for other image contents or styles, especially if color plays a substantial role in the artwork (e.g., in “Kitsch” artworks, Pelowski et al., 2020). Other aspects of viewers may also have important modulating influences (e.g., Leder et al., 2004). We expect that measuring the current emotional state not only before briefly viewing an image but also before viewing it for an unlimited amount of time could have further contributed to answering the questions in our study. Further limitations to our study may include the homogeneous sample – mostly female participants aged 20–25 took part in our experiment. As it is known that women have a slight preference for pastel colors, a study with more male participants could provide further insights on the effects of color manipulation, given men’s preference for saturated colors (Palmer and Schloss, 2015).

Moreover, while comparable to other studies in empirical aesthetics (Locher et al., 1999; Boust and Ezrati, 2006; Siri et al., 2018), the number of images was kept relatively low to minimize participant burden. This trade-off resulted in lower statistical power, which may increase the risk of a Type II error (i.e., false negatives). Future studies should increase their statistical power by using more images and recruiting more participants to assess whether our results can be replicated and whether our study overlooked specific effects.

In line with previous works (Valdez and Mehrabian, 1994; Camgöz et al., 2002; Palmer and Schloss, 2015), we expected that increasing saturation would increase liking. We also assumed that this effect would be more pronounced for laypersons, whereas experts would be more influenced by the content of the image (Commare et al., 2018; Pelowski et al., 2020). That is why we selected images of paintings with a muted color palette and increased their color saturation. For future work, it would be interesting to examine whether images that originally have very saturated colors are liked less when the saturation is reduced.

Next, while we asked participants to indicate at the end of the study whether any of the presented images were familiar to them, we did not measure (perceived) familiarity. Future studies on the effects of color saturation should consider including familiarity as a covariable, as it is a known predictor of image liking (Leder et al., 2004).

In our study, we wanted to investigate the effect of altered saturation as an image feature that often varies unintentionally in digitally reproduced art images on the Internet. In that context, however, images are rarely seen in isolation. The isolated presentation of images in the present study may thus be seen as a limitation in recreating the real situation of how images are seen on the Internet. To investigate the effect that juxtaposed images have on each other is the content of our next study.

In summary, it can be said that whenever a digitized artwork is downloaded from the Internet, the choice of a single version of color saturation out of countless variations exerts an influence on the reception of the image and thus needs to be controlled carefully.

## Data Availability Statement

The raw data supporting the conclusions of this article will be made available by the authors, without undue reservation.

## Ethics Statement

Ethical review and approval was not required for the study on human participants in accordance with the local legislation and institutional requirements. The patients/participants provided their written informed consent to participate in this study.

## Author Contributions

CR, MP, KO, and EM contributed to conceiving and designing the study. TT carried out the measurement of color saturation on the image stimuli. CR prepared the study and collected the survey data. CR and EM performed the statistical analysis. CR, MP, and EM wrote the first draft of the manuscript and wrote the sections of the manuscript. All authors contributed to the article and approved the submitted version.

### Conflict of Interest

The authors declare that the research was conducted in the absence of any commercial or financial relationships that could be construed as a potential conflict of interest.

## Footnotes

^1^https://kunstmuseumbasel.ch/en/collection/collectiononline

^2^The color conformity of the digital reproductions with the original works was checked. The images owned by the Kunstmuseum Basel were evaluated by the authors themselves in terms of color conformity; for the remaining images, the individual museums or auction houses were asked to confirm the color correspondence.
