# Supplementary material for: Aesthetic Evaluation of Digitally Reproduced Art Images
Source: Front Psychol. 2020 Dec 11;11:615575. doi: 10.3389/fpsyg.2020.615575 (PMC7759521; doi:10.3389/fpsyg.2020.615575)
Supplement: Supplementary file 1 [file Data_Sheet_1.pdf]

Supplementary Material

Table A1: List of all used artworks

Artist	Year	Title	Museum	Image Specifications	Information on saturation manipulation	Link	Genre
Camille Pissarro	1900	Bouquet de fleurs	Kunst-museum Basel	Oil on silk on canvas 18 x 14.1 cm	+70%	http://sammlungonline.kunstmuseumbasel.ch/eMuseumPlus?service=direct/1/ResultDetailView/result.inline.list.t1.collection_list.$TspTitleLink.link&sp=13&sp=Sartist&sp=SfilterDefinition&sp=0&sp=27&sp=1&sp=SdetailView&sp=39&sp=Sdetail&sp=0&sp=T&sp=0&sp=SdetailList&sp=0&sp=F&sp=Scollection&sp=l2066	Stillife
Henri Matisse	1920	Femme au divan	Kunst-museum Basel	Oil on canvas 60.2 x 73.4 cm	+70%	https://kmb.pictupark.com/Go/4CVqeqEs	Figures
Edouard Vallet	1912	Autoportrait	Kunst-museum Basel	Oil on canvas 44 x 37 cm	+60%	http://sammlungonline.kunstmuseumbasel.ch/eMuseumPlus?service=direct/1/ResultDetailView/result.inline.list.t1.collection_list.$TspTitleImageLink.link&sp=13&sp=Sartist&sp=SfilterDefinition&sp=0&sp=11&sp=1&sp=SdetailView&sp=2&sp=Sdetail&sp=0&sp=T&sp=0&sp=SdetailList&sp=0&sp=F&sp=Scollection&sp=l1319	Portrait

Ferdinand Hodler	1904	Bildnis Käthe von Bach (im Garten)	Kunst-museum Basel	Oil on canvas 42.2 x 32.6 cm	+60%	http://sammlungonline.kunstmuseumbasel.ch/eMuseumPlus?service=direct/1/ResultDetailView/result.inline.list.t1.collection_list.$TspTitleLink.link&sp=13&sp=Sartist&sp=SfilterDefinition&sp=0&sp=27&sp=1&sp=SdetailView&sp=68&sp=Sdetail&sp=0&sp=T&sp=0&sp=SdetailList&sp=0&sp=F&sp=Scollection&sp=l1190	Portrait
Max Birrer	1934	Oranges amères	Kunst-museum Basel	Oil on canvas 46.5 x 61 cm	+70%	http://sammlungonline.kunstmuseumbasel.ch/eMuseumPlus?service=direct/1/ResultDetailView/result.inline.list.t1.collection_list.$TspTitleImageLink.link&sp=13&sp=Sartist&sp=SfilterDefinition&sp=0&sp=7&sp=1&sp=SdetailView&sp=91&sp=Sdetail&sp=0&sp=T&sp=0&sp=SdetailList&sp=0&sp=F&sp=Scollection&sp=l3155	Stillife
Edvard Munch	1918	Küsten-landschaft	Kunst-museum Basel	Oil on canvas 120.9 x 160 cm	+60%	http://sammlungonline.kunstmuseumbasel.ch/eMuseumPlus?service=direct/1/ResultDetailView/result.inline.list.t1.collection_list.$TspTitleImageLink.link&sp=13&sp=Sartist&sp=SfilterDefinition&sp=0&sp=8&sp=1	Land-scape

						&sp=SdetailView&sp=234&sp=Sdetail&sp=0&sp=T&sp=0&sp=SdetailList&sp=0&sp=F&sp=Scollection&sp=l1250	
Paul Gaugin	1892	Ta matete (Le marché)	Kunst-museum Basel	Oil on jute 73.2 x 91.5 cm	+70%	http://sammlungonline.kunstmuseumbasel.ch/eMuseumPlus?service=direct/1/ResultDetailView/result.inline.list.t1.collection_list.$TspTitleLink.link&sp=13&sp=Sartist&sp=SfilterDefinition&sp=0&sp=10&sp=1&sp=SdetailView&sp=7&sp=Sdetail&sp=0&sp=T&sp=0&sp=SdetailList&sp=0&sp=F&sp=Scollection&sp=l1162	Figures
Paul Cézanne	1885/1887	Cinq baigneuses	Kunst-museum Basel	Oil on canvas 65.3 x 65.3 cm	+70%	http://sammlungonline.kunstmuseumbasel.ch/eMuseumPlus?service=direct/1/ResultDetailView/result.inline.list.t1.collection_list.$TspTitleLink.link&sp=13&sp=Sartist&sp=SfilterDefinition&sp=0&sp=3&sp=1&sp=SdetailView&sp=32&sp=Sdetail&sp=0&sp=T&sp=0&sp=SdetailList&sp=0&sp=F&sp=Scollection&sp=l1136	Figures
Amedeo Modigliani	1918	Marie (Marie, fille du peuple)	Kunst-museum Basel	Oil on canvas 61.2 x 49.8 cm	+60%	http://sammlungonline.kunstmuseumbasel.ch/eMuseumPlus?service=direct/1/ResultDetailView/result.inline.list.t1.c	Portrait

						ollection_list.$TspTitleLink.link&sp=13&sp=Sartist&sp=SfilterDefinition&sp=0&sp=21&sp=1&sp=SdetailView&sp=184&sp=Sdetail&sp=0&sp=T&sp=0&sp=SdetailList&sp=0&sp=F&sp=Scollection&sp=l1244	
Vincent van Gogh	1889	Vue de l'asile et de la Chapelle de Saint-Rémy	Private Collection	Oil on Canvas 45.1 x 60.4 cm	+70%	https://www.christies.com/lotfinder/Lot/vincent-van-gogh-1853-1890-vue-de-5532359-details.aspx	Land-scape
Egon Schiele	1910	Stadt am blauen Fluss (Krumau)	Private Collection	Gouache, watercolor, metallic paint and black Conté crayon on paper 45 x 31.4 cm	+50	https://commons.wikimedia.org/wiki/File:Egon_Schiele_-_Stadt_am_blauen_Fluss_(1910).jpg	Land-scape
Claude Monet	1871	La Tamise à Westminster	National Gallery London	Oil on canvas 47 x 73 cm	+60%	https://www.nationalgallery.org.uk/paintings/claude-monet-the-thames-below-westminster	Land-scape
Paula Modersohn-Becker	1907	Stilleben mit Ringelrosen und Bernsteinkette vor Landschaft	Private Collection	Oil on canvas 25.0 x 28.5 cm	+50%	https://www.museen-boettcherstrasse.de/ausstellungen/paula-modersohn-becker-aus-privaten-sammlungen/	Stillife
Paul Cézanne	1877	Nature morte avec pot, tasse et pommes	Metropolitan Museum of Art, New York	Oil on canvas 60.6 x 73.7 cm	+80%	https://www.metmuseum.org/toah/works-of-art/29.100.66/	Stillife
Vincent van Gogh	1890	La cour de la prison	Pushkin Museum of Fine Arts, Moscow	Oil on canvas 80 x 64 cm	+60%	https://de.wikipedia.org/wiki/Datei:Vincent_Willem_van_Gogh_037.jpg	Figures

Alexej von Jawlensky	1913	Grosser Frauenkopf auf Rot	Pinakothek der Moderne München	Oil on cardboard 68.8 x 49.9 cm	+60%	https://www.sammlung.pinakothek.de/de/bookmark/artwork/bwx0338Y4m	Portrait

Table A2: Overall saturation and brightness of the used stimulus images

	**Saturation** original	manipulated	increase	**Brightness** original	manipulated	increase
Birrer "Oranges ameres"	41	96	133%	142	161	14%
Cezanne "Baigneuses"	66	152	131%	139	173	24%
Cezanne "Nature morte"	87	179	105%	106	136	28%
Gaugin "Te matete"	106	184	74%	128	159	24%
Hodler "Käthe"	66	131	99%	160	188	18%
Jawlensky "Frauenkopf "	94	175	85%	95	123	29%
Matisse "Femme au divan"	57	129	127%	161	192	19%
Modersohn "Stillleben"	66	109	65%	137	153	11%
Modigliani "Marie"	105	161	53%	102	122	20%
Monet "Themse"	41	89	118%	168	188	12%
Munch "Küstenlandschaft"	66	127	94%	120	141	17%
Pissarro "Bouquet"	67	152	127%	108	137	26%
Schiele "Stadt"	86	143	67%	125	147	18%
Vallet "Autoportrait"	86	156	82%	123	148	20%
Van Gogh "La cour"	38	82	113%	136	149	10%
Van Gogh "Vue de l'asile"	82	176	116%	143	185	29%
Averages	72	140	99%	131	156	20%

Table A3: Descriptive Statistics for all used images

Set 1

Paul Cézanne, Cinque baigneuses, 1885/1887										
		Original					Augmented saturation				
	n	*Mean*	*Median*	*SD*	*Min*	*Max*	*Mean*	*Median*	*SD*	*Min*	*Max*
100ms	72	43.76	46	24.541	0	93	44.17	45	24.888	0	93
Unrestricted time	72	46.72	45	24.470	0	96	41.10	40	24.169	0	96

Paul Gaugin, Te matate (le marché), 1892										
		Original					Augmented saturation				
	n	*Mean*	*Median*	*SD*	*Min*	*Max*	*Mean*	*Median*	*SD*	*Min*	*Max*
100ms	72	42.56	42	24.310	0	95	44.26	40	24.470	0	95
Unrestricted time	72	53.46	55	22.259	11	100	52.54	55	23.855	0	90

Amedeo Modigliani, Marie, 1918											
		Original					Augmented saturation				
	n	*Mean*	*Median*	*SD*	*Min*	*Max*	*Mean*	*Median*	*SD*	*Min*	*Max*
100ms	72	49.49	49.50	27.740	4	100	47.79	41.50	27.587	4	100
Unrestricted time	72	47.40	46	27.519	0	100	47.68	50	28.294	0	100

Edouard Vallet, Autoportrait, 1912											
		Original					Augmented saturation				
	n	*Mean*	*Median*	*SD*	*Min*	*Max*	*Mean*	*Median*	*SD*	*Min*	*Max*
100ms	72	39.85	38.50	25.943	0	86	38.57	35	25.323	0	85
Unrestricted time	72	40.08	40	24.338	0	100	41.14	40	24.359	0	100

Claude Monet, La Tamise à Westminster, 1871											
		Original					Augmented saturation				
	n	*Mean*	*Median*	*SD*	*Min*	*Max*	*Mean*	*Median*	*SD*	*Min*	*Max*
100ms	72	59.79	62	23.214	0	95	59.82	65	25.789	0	98
Unrestricted time	72	59.75	66	23.558	0	96	65.83	71.50	24.508	0	100

Edvard Munch, Küstenlandschaft, 1918											
		Original					Augmented saturation				
	n	*Mean*	*Median*	*SD*	*Min*	*Max*	*Mean*	*Median*	*SD*	*Min*	*Max*
100ms	72	48.21	49.50	26.146	0	100	50.33	53.50	25.031	0	100
Unrestricted time	72	56.10	59	24.252	0	100	58.03	60	25.548	0	100

Camille Pissarro, Bouquet de fleurs, 1900											
		Original					Augmented saturation				
	n	*Mean*	*Median*	*SD*	*Min*	*Max*	*Mean*	*Median*	*SD*	*Min*	*Max*
100ms	72	40.13	43	22.457	0	100	44.21	46	25.682	0	100
Unrestricted time	72	40.96	43	25.891	0	95	47.38	48	23.753	0	89

Max Birrer, Oranges amères, 1934											
		Original					Augmented saturation				
	n	*Mean*	*Median*	*SD*	*Min*	*Max*	*Mean*	*Median*	*SD*	*Min*	*Max*
100ms	72	45.78	50	23.632	0	100	48.85	50	24.337	0	100
Unrestricted time	72	48.25	50	24.218	0	100	54.18	58.50	24.823	5	90

Set 2

Henri Matisse, Femme au divan, 1920										
		Original					Augmented saturation			
	n	*Mean*	*Median*	*SD*	*Min*	*Max*	*Mean*	*Median*	*SD*	*Min*	*Max*
100ms	75	43.76	46	24.541	0	93	44.17	45	24.888	0	93
Unrestricted time	75	46.72	45	24.470	0	96	41.10	40	24.169	0	96

Vincent van Gogh, La cour de la prison, 1890											
		Original					Augmented saturation				
	n	*Mean*	*Median*	*SD*	*Min*	*Max*	*Mean*	*Median*	*SD*	*Min*	*Max*
100ms	75	49.05	51	21.843	0	91	48.33	49	20.958	0	90
Unrestricted time	75	57.43	60	23.982	0	100	60.19	60	24.543	0	100

Ferdinand Hodler, Bildnis Käthe von Bach (im Garten), 1904											
		Original					Augmented saturation				
	n	*Mean*	*Median*	*SD*	*Min*	*Max*	*Mean*	*Median*	*SD*	*Min*	*Max*
100ms	75	56.67	58	23.131	2	100	58.59	60	23.515	0	100
Unrestricted time	75	58.91	65	27.296	4	100	59.33	65	24.843	0	100

Alexej von Jawlensky, Grosser Frauenkopf auf Rot, 1913											
		Original					Augmented saturation				
	n	*Mean*	*Median*	*SD*	*Min*	*Max*	*Mean*	*Median*	*SD*	*Min*	*Max*
100ms	75	49.85	52	25.083	0	100	49.96	48	25.252	0	100
Unrestricted time	75	46.19	45	23.316	0	96	48.92	54	25.700	0	94

Vincent van Gogh, Vue de l'asile et de la Chapelle de Saint-Rémy, 1889											
		Original					Augmented saturation				
	n	*Mean*	*Median*	*SD*	*Min*	*Max*	*Mean*	*Median*	*SD*	*Min*	*Max*
100ms	75	56.04	62	22.702	0	100	50.97	50	23.267	5	100
Unrestricted time	75	56.36	56	25.396	6	100	50.39	51	28.349	0	100

Egon Schiele, Stadt am blauen Fluss (Krumau), 1910											
		Original					Augmented saturation				
	n	*Mean*	*Median*	*SD*	*Min*	*Max*	*Mean*	*Median*	*SD*	*Min*	*Max*
100ms	75	49.00	50	23.684	1	100	50.75	52	21.467	3	100
Unrestricted time	75	45.24	45	23.718	0	94	44.25	40	27.224	0	95

Paula Modersohn-Becker, Stilleben mit Ringelrosen und Bernsteinkette vor Landschaft, 1907											
		Original					Augmented saturation				
	n	*Mean*	*Median*	*SD*	*Min*	*Max*	*Mean*	*Median*	*SD*	*Min*	*Max*
100ms	75	43.00	43	23.417	0	97	43.79	43	23.446	0	90
Unrestricted time	75	32.52	25	23.294	0	91	39.65	38	24.916	0	100

Paul Cézanne, Nature morte avec pot, tasse et pommes, 1877											
		Original					Augmented saturation				
	n	*Mean*	*Median*	*SD*	*Min*	*Max*	*Mean*	*Median*	*SD*	*Min*	*Max*
100ms	75	43.97	41	23.974	4	100	45.79	46	23.780	5	90
Unrestricted time	75	38.25	37	24.113	0	100	36.61	33	21.558	0	88
